# Effects of Zinc Methionine Hydroxy Analog Chelate on Laying Performance, Serum Hormone Levels, and Expression of Reproductive Axis Related Genes in Aged Broiler Breeders

**DOI:** 10.3389/fvets.2022.918283

**Published:** 2022-07-04

**Authors:** Bowen Yang, Jiangang Gong, Jialin Jing, Yanshuang Hao, Shupeng Li, Guanzhong Liu, Zhihua Feng, Guoxian Zhao

**Affiliations:** ^1^College of Animal Science and Technology, Hebei Agricultural University, Baoding, China; ^2^College of Food Science and Technology, Hebei Agricultural University, Baoding, China

**Keywords:** Zinc methionine hydroxy analog chelate, laying performance, hormone levels, reproduction, gene expression, broiler breeder

## Abstract

Inorganic zinc (Zn) supplements are commonly used in poultry feeds, but their low utilization results in the increase of Zn excretion. Thus, to provide a new perspective for the substitution of inorganic Zn, a novel Zn methionine hydroxy analog chelate (Zn-MHA) was studied in the present study to evaluate its effects on laying performance, serum hormone indexes and reproductive axis-related genes in broilers breeders. A total of 480 Hubbard breeders (56-week-old) were fed a basal diet (containing 27.81 mg Zn/kg) without Zn addition for 2 weeks, and then allocated to 4 groups with 6 replicates (each replicate consisting of 10 cages and 2 breeders per cage) for 10 weeks. Four treatment diets given to broiler breeders included the basal diet added with 25, 50, and 75 mg/kg of Zn-MHA and 100 mg/kg of Zn sulfate (ZnSO_4_). The laying rate, egg weight and feed conversation ratio increased in the 75 mg/kg Zn-MHA group compared to the ZnSO_4_ group. The eggshell thickness was not decreased with the addition of 50 mg/kg and 75 mg/kg Zn-MHA in the diet compared to the 100 mg/kg ZnSO_4_ group. There was a significant improvement in the reproductive performance of breeders in the 75 mg/kg Zn-MHA group, including the fertility and 1-day-old offspring weight. Besides, serum sex hormone levels including FSH and P_4_ increased significantly in 75 mg/kg Zn-MHA group. No significant effect on the ovarian weight or the number of follicles in broiler breeders was observed by supplementing Zn-MHA. Compared to the 100 mg/kg ZnSO_4_ group, dietary supplementation with 75 mg/kg of Zn-MHA showed an up-regulation of the *FSHR* mRNA in the granular layer of follicles. However, dietary supplementation of Zn-MHA had no effects on mRNA expressions of the ovarian *LHR* and *PRLR* genes. These findings reinforce the suggestion that Zn-MHA (75 mg/kg) could replace ZnSO_4_ (100 mg/kg) as a Zn supplement in diet of broiler breeders, which resulted in better laying and reproduction performances by regulating the expression levels of reproductive axis related genes and serum hormone levels.

## Introduction

Zinc (Zn) is an essential trace element that participates in the composition of numerous enzymatic systems and has various functions, involving energy, carbohydrate, nucleic acid and protein metabolism (1). In the poultry industry, Zn plays a critical role in laying performance, egg quality (2), reproductive performance (3), and antioxidant activity (4). In the plant-feeding ingredients, Zn is often complexed with phytic acid, thereby hindering its absorption (5). Therefore, additional Zn supplements are usually required to meet the Zn need in the poultry industry. Inorganic Zn supplements, such as ZnO and ZnSO_4_, are the most commonly used zinc supplements in poultry feed due to their low price and easy preparation. However, adversary effects of inorganic Zn, such as low bioavailability, large excretion, high degree of oxidation, and destruction of nutrition, have been criticized by poultry farming practitioners (6). Zn often competes with other trace elements or is inhibited by antagonists, which affects its bioavailability (7). Therefore, finding a pathway that is not affected by other trace elements or inhibitors will be the key to improving Zn utilization.

Zinc methionine hydroxy analog chelate (Zn-MHA), a newly designed Zn fortifier, has a ring structure like other Zn amino acid chelates. The two deprotonated methionine hydroxy analog (MHA) molecules coordinate with the metal cations through the two oxygen atoms of the carboxyl and hydroxyl groups, respectively, to form two penta-atomic chelate rings (8). The special ring structure that surrounds Zn ions protects them from the inhibitors (9). The MHA, also known as 2-hydroxy-4-(methylthio) butanoic acid, is a precursor used to supplement methionine (10). MHA has higher bioavailability than DL-methionine in poultry (11). Importantly, MHA can form stable chelates with divalent metals. The preparation of Zn-MHA is to put MHA and metal inorganic salt aqueous solution in the presence of alkaline solution to maintain a certain reaction temperature (8). Therefore, compared with other amino acid chelates, Zn-MHA has the advantages of simple preparation and lower price. The biosafety of MHA metal chelate was verified by human intestinal CACO-2 cells *in vitro* (8). Previous study in rats showed that Zn-MHA had higher bioavailability than ZnSO_4_, which was manifested as decreased Zn excretion and increased hepatic Zn deposition (12). Similarly, Zn methionine (Zn-Met) has been proved to be a more available source of Zn than inorganic forms like ZnO or ZnSO_4_ (13). Zn-Met declines the antagonistic interactions between Zn and nutrients or non-nutritive components in the gastrointestinal tract, which enhances the bioavailability of Zn (14, 15).

Zn is of great significance to ensure the laying performance and egg quality of breeders (16). In the late phase of egg-laying, laying performance and thickness of eggshells are poor, especially the expensive breeding eggs will bring greater economic losses (17). Therefore, finding a way to improve the utilization rate of Zn in the diet will be an effective way to ensure the performance of broiler breeders in the late phase. The effects of Zn-MHA on broiler breeders during the late phase are poorly known. Hence, the effects of total replacement of inorganic Zn by Zn-MHA on performance, serum hormone levels, and expression of reproductive axis related genes of aged broiler breeders were evaluated in the present study.

## Materials and Methods

### Animals, Diets, and Design

The experimental Zn-MHA (16% of Zn and 80% of methionine) was provided by Novus Inc. (St. Charles, MO, USA). A total of 480 (56-week-old) Hubbard breeding hens with similar body weight and initial egg production were housed in cages and randomly assigned to 4 treatment groups with 6 replicates. Each replicate contained 10 cages and 2 breeders per cage. Four treatment diets given to broiler breeders were as follows: basal diet supplemented with 100 mg/kg ZnSO_4_ as control group; basal diet with 25, 50, and 75 mg/kg Zn-MHA as three trail groups, respectively. The available Zn contents in the four experimental diets were: 129.80, 53.20, 80.71, and 108.60 mg/kg, respectively. Each breeder was fed a fixed diet of 158 g/d. During the experiment, water was free to access for the breeders. The feeding trial lasted for 10 weeks, after being fed a basal Zn diet (27.81 mg/kg) without extra Zn for 2 weeks to deplete the Zn in breeders. The basal diet was formulated with reference to NRC (1994). Methionine was supplemented in the premix for each treatment group to eliminate nutrient imbalances. The basal diet formula and nutrients are listed in Table 1. During the experiment, breeders were housed under a controlled temperature (25 ± 2°C) and relative humidity (74 ± 10%). Regular lighting (16L:8D) and ventilation were also performed throughout the experiments.

**Table 1:** Dietary composition and nutrient levels of the basal diet for broiler breeders (air-dried basis).

**Ingredients (%)**	
Corn	59.15
Soybean meal	28.00
Soybean oil	3.28
Choline chloride (98%)	0.20
Limestone	8.66
Methionine	0.17
Threonine	0.04
Premix^a^	0.50
Total	100.00
**Nutrients^b^**	
Crude protein (%)	14.60
Metabolizable energy (MJ/kg)	11.72
Calcium (%)	3.60
Total phosphorus (%)	0.53
Available phosphorus (%)	0.32
Digestible Methionine (%)	0.42
Digestible Lysine (%)	0.71
Zn (mg/kg)	27.81

a *Supplied per kg diet: vitamin A, 10,000 IU; vitamin D_3_, 3,500 IU; vitamin E, 100 IU; vitamin K, 3 mg; thiamine, 3 mg; riboflavin, 6 mg; pantothenic acid, 15 mg; niacin, 35 mg; pyridoxine, 3 mg; biotin, 0.15 mg; folic acid, 1.5 mg; vitamin B_12_, 0.02 mg; Mn, 120 mg; I, 1.25 mg; Fe, 40 mg; Cu, 16 mg; Se, 0.3 mg*.

b *The values are analyzed values*.

### Sample Preparation

Egg numbers and egg weight were measured every day at a fixed time during the feeding trial. On the last day of the feeding trial, 5 eggs per replicate were chosen for quality determination. One hen was randomly chosen from each replicate for blood collection. Blood was taken from the vein under the wings into a coagulation-promoting tube. After centrifugation at 1,500 × *g* at 4°C for 15 min, serum was collected. Three hens from each treatment were then killed by cervical dislocation. Ovaries were removed and weighed. Follicles were classified as small yellow follicles (SYFs, 4 to 10 mm) and large white follicles (LWFs, 2 to 4 mm) according to their size. All types of follicles in the ovary were counted after sorting by size. The first three largest preovulatory follicles (POFs) were removed from the ovary and were designated as F1, F2, and F3 (18). The granulosa layers of SYFs, LWFs, and POFs were collected and frozen by liquid nitrogen and stored at −80°C.

### Laying Performance and Egg Quality

The laying rate was calculated based on the daily records, whereas the feed conversion rate (FCR) was calculated weekly and was expressed as feed to egg weight.

The egg quality indexes included shape index, eggshell strength, eggshell thickness, yolk color, yolk weight, albumen height, and Haugh unit. The shape index was calculated by dividing egg width by egg length. Eggshell strength was evaluated performed by Egg Force Reader. The albumen height, yolk color, and Haugh unit were measured using an egg analyzer. The above two instruments used were procured from Orka Ltd. (Ramat HaSharon, Israel). Eggshell thickness was measured by a vernier caliper.

### Hatching Performance

To evaluate the hatching traits, eggs were incubated in week 8 to 10. A total of 15 clean eggs without visible abnormalities were selected for incubation of each replicate per week. The eggs were stored in a controlled environment room for 7 d and the temperature was maintained at 18 to 20°C with a relative humidity of 75% to 80%. The incubation was then conducted in a commercial multi-stage incubator (YALN-19200, Jinan, China) at 37.5°C with 60% relative humidity. Unfertilized eggs were removed following candling on day 7 and 18 of the incubation were also examined to pick out non-viable eggs during incubation. The reproductive performance included fertility, hatchling capacity, hatchling capacity of fertile eggs, total embryonic mortality, healthy offspring percentage, and 1-day-old offspring weight.

### Serum Hormone Analysis

The concentrations of follicle-stimulating hormone (FSH), luteinizing hormone (LH), prolactin (PRL), estradiol (E_2_), progesterone (P_4_), triiodothyronine (T_3_) and thyroxine (T_4_) were determined by assay kits obtained from J&L Biotechnology Technology Co., Ltd (Shanghai, China).

### mRNA Relative Expression Levels of Reproductive Axis Genes by Real-Time Quantitative PCR

The determination of mRNA relative expression levels including follicle-stimulating hormone receptor (*FSHR*), luteinizing hormone receptor (*LHR*), and prolactin receptor (*PRLR*) were conducted by the SYBR Green I Real-time qPCR analysis. Total RNA was extracted from granulosa layer samples, using Total RNA extraction kit (GenePool Biotech Co., Ltd., Beijing, China). The output of RNA was measured by a spectrophotometer (Thermo Scientific, Wilmington, DE, USA), and electrophoresis was used to evaluate its quality.

Quantification was performed by reverse transcription (RT) and PCR. Following the technical manual of the mRNA-cDNA synthesis kit (GenePool Biotech Co., Ltd., Beijing, China), each RT reaction consisted of 1 μg RNA, 4 μL of 5 × RT Buffer, 4 μL of dNTP Mix (2.5 mM each), 1 μL of total RNA, 2 μL of DTT (0.1 M), 1 μL of HiFiScript (200 U/μL), and 2 μL of Primer Mix, and adding RNase-free water to 20 μL. Reactions were performed for 50 min at 42°C, followed by heat inactivation of reaction for 5 min at 85°C. The 20 μL RT reaction mix was kept at −20°C.

Analysis of each sample was performed in triplicate. The primers were synthesized by GenePool Biotech Co., Ltd. (Beijing, China) according to previous study (19), as shown in Table 2. Real-time qPCR was conducted using a LineGene 9600 Plus (Bioer Technology Co. Ltd., Hangzhou, China) PCR system with a total volume of 20 μL containing 2 μL of cDNA, 10 μL of 2 × fast SYBR mixture, 0.4 μL of forward primer, 0.4 μL of reverse primers, and 7.2 μL of nuclease-free water. Reaction mixtures were incubated at 95°C for 10 min, followed by 40 cycles at 95°C for 10 s, and 60°C for 30 s. After the PCR cycles, melting curve analysis was carried out to confirm the specific generation of the PCR products.

**Table 2:** Sequences of real-time qPCR primers.

**Gene^1^**	**Primer**	**Sequence**	**Product length** **(bp)**	**Annealing temperature** **(°C)**	**GenBank accession number**
*FSHR*	Forward (5′-3′)	TCAGCAGCTACATGAAGGT	103	60	NM 205079.1
	Reverse (3′-5′)	AAGGCAAGTACATTCAACACTA			
*LHR*	Forward (5′-3′)	GCTGATCTTAATGCTCAACG	120	60	NM 204936.1
	Reverse (3′-5′)	TTGGCAATCTTGGTGTCTTTAT			
*PRLR*	Forward (5′-3′)	CAGATTCACGAGTTCCGCA	172	60	NM 204854.1
	Reverse (3′-5′)	GGCATAAATGAGGATGGGT			
β-actin	Forward (5′-3′)	ACGTCGCACTGGATTTCGAG	282	60	NM 205518.1
	Reverse (3′-5′)	TGTCAGCAATGCCAGGGTAC			

1 *FSHR, follicle-stimulating hormone receptor; LHR, luteinizing hormone receptor; PRLR, prolactin receptor; β-actin, avian β-actin*.

The relative expression of the mRNA was normalized to the expression of β-actin, and the 2^−Δ*ΔCt*^ method was used to calculate the levels of relative expressions (20).

### Statistical Analysis

The difference between the control (ZnSO_4_) and each experimental group was carried out using the independent-samples *t* test by statistical analysis system (SAS Institute Inc., Cary, NC, USA). The orthogonal contrast was performed to test the linear and quadratic *P*-values of increasing dietary Zn-MHA levels. *P* < 0.05 was considered to be significant. The replicate was the experimental unit for the laying performance. The results are expressed as mean and standard deviation.

## Results

### Effect of Zn-MHA on Laying Performance of Broiler Breeders

It can be seen from Table 3 that the laying rate and average egg weight increased linearly and quadratic (*P* < 0.001) by increasing the concentration of Zn-MHA, and reached the highest in the group fed with a 75 mg/kg Zn-MHA diet. Compared to the ZnSO_4_ group, dietary supplementation of 75 mg/kg Zn-MHA increased the laying rate (*P* < 0.05). However, 25 mg/kg Zn-MHA group showed a decrease in laying rate compared to the ZnSO_4_ group. We found that dietary supplementation of 50 and 75 mg/kg Zn-MHA showed an improvement both in egg weight and FCR value (*P* < 0.05). The broken egg rate was not affected by whatever source of Zn (*P* > 0.05).

**Table 3:** Effects of dietary Zn-MHA supplementation on laying performance of broiler breeders^a^.

**Items**	**Dietary Zn supplementation**				***P*-value**	
	**100 mg/kg ZnSO_**4**_**	**Zn-MHA (mg/kg)**				
		**25**	**50**	**75**	**Linear**	**Quadratic**
Laying rate (%)	58.50 ± 0.14	57.17 ± 0.17^*^	59.00 ± 0.30	58.83 ± 0.22^*^	<0.001	<0.001
Average egg weight (g)	67.50 ± 0.37	67.67 ± 0.38	68.17 ± 0.39^*^	68.50 ± 0.42^**^	0.002	0.007
FCR (feed/egg)	4.00 ± 0.03	4.07 ± 0.02^*^	3.94 ± 0.03^**^	3.92 ± 0.03^**^	<0.001	<0.001
Broken egg rate (%)	4.64 ± 0.96	3.79 ± 0.84	4.79 ± 1.40	3.97 ± 1.44	0.809	0.373

*FCR, Feed conversion ratio; Zn-MHA, Zinc methionine hydroxy analog chelate*.

a *Data represent mean ± SD values of 6 replicates each treatment*.

* *refers to P < 0.05 and*

** *refers to P < 0.01 between the control and experimental groups by independent-sample t test*.

### Effect of Zn-MHA on Egg Quality of Broiler Breeders

Dietary Zn-MHA supplementation had no effects on egg quality of breeders at the end of the trial based on the data of shape index, yolk color, yolk weight, albumen height, Haugh unit or eggshell strength compared with the ZnSO_4_ group (*P* > 0.05; Table 4). The eggshell thickness decreased in the 25 mg/kg Zn-MHA group in the diet (*P* < 0.05). However, the eggshell thickness of 50 mg/kg and 75 mg/kg Zn-MHA group did not differ with the control group.

**Table 4:** Effects of dietary Zn-MHA supplementation on egg quality of broiler breeders^a^.

**Items**	**Dietary Zn supplementation**				***P*-value**	
	**100 mg/kg ZnSO_**4**_**	**Zn-MHA (mg/kg)**				
		**25**	**50**	**75**	**Linear**	**Quadratic**
Shape index	1.35 ± 0.03	1.37 ± 0.03	1.37 ± 0.04	1.34 ± 0.02	0.103	0.159
Yolk color	8.17 ± 0.89	8.89 ± 1.05	8.50 ± 0.81	8.67 ± 0.67	0.653	0.737
Yolk weight (g)	22.76 ± 1.29	23.37 ± 0.66	22.61 ± 1.03	24.15 ± 1.85	0.340	0.148
Eggshell strength (N)	34.83 ± 2.97	33.67 ± 1.26	34.83 ± 1.01	35.33 ± 0.94	0.014	0.047
Eggshell thickness (mm)	0.36 ± 0.01	0.34 ± 0.01^*^	0.36 ± 0.01	0.36 ± 0.01	0.002	<0.001
Albumen height (mm)	5.16 ± 0.78	5.24 ± 0.87	5.44 ± 0.78	5.12 ± 0.72	0.784	0.779
Haugh unit	65.55 ± 7.31	66.11 ± 7.26	66.28 ± 7.90	67.71 ± 5.40	0.980	0.924

*Zn-MHA, Zinc methionine hydroxy analog chelate*.

a *Data represent mean ± SD of 6 replicates each treatment*.

* *refers to P < 0.05 between the control and experimental groups by independent-sample t test*.

### Effect of Zn-MHA on Hatching Performance of Broiler Breeders

As shown in Table 5, there was a significant improvement in the fertility rate of the broilers belonging to the 75 mg/kg Zn-MHA diet-fed group compared to the ZnSO_4_ group (*P* < 0.05). No effects of dietary Zn-MHA supplementation on hatchling capacity, hatching capacity of fertile eggs, percentage of healthy offspring, and total embryonic mortality were observed. Compared to the ZnSO_4_ group, the offspring weight of the 50 and 75 mg/kg Zn-MHA diet fed groups was significantly increased (*P* < 0.05).

**Table 5:** Effects of dietary Zn-MHA supplementation on hatching performance in broiler breeders^a^.

**Items**	**Dietary Zn supplementation**				***P*-value**	
	**100 mg/kg ZnSO_**4**_**	**Zn-MHA (mg/kg)**				
		**25**	**50**	**75**	**Linear**	**Quadratic**
Egg number in the incubator	90	90	90	90	-	-
Fertility (%)	89.59 ± 0.90	89.70 ± 0.37	91.05 ± 0.60	91.78 ± 1.21^*^	0.012	0.049
Hatchling capacity (%)	76.23 ± 4.61	75.20 ± 5.65	74.90 ± 4.60	76.38 ± 4.25	0.760	0.927
Hatching capacity of fertile eggs (%)	81.92 ± 1.08	82.37 ± 2.72	81.26 ± 1.24	82.62 ± 2.46	0.890	0.739
Healthy offspring (%)	95.17 ± 1.10	95.42 ± 1.47	95.16 ± 0.16	96.00 ± 1.46	0.555	0.696
Total Embryonic mortality (%)	1.80 ± 0.00	1.60 ± 0.40	1.80 ± 0.60	1.50 ± 0.30	0.788	0.722
Offspring weight (g)	45.56 ± 0.74	45.80 ± 0.86	48.02 ± 0.21^*^	47.78 ± 1.34^*^	0.060	0.049

*Zn-MHA, Zinc methionine hydroxy analog chelate*.

a *Data represent mean ± SD of 90 eggs each treatment*.

* *refers to P < 0.05 between the control and experimental groups by independent-sample t test*.

### Effect of Zn-MHA on Serum Hormone of Broiler Breeders

Dietary supplementation of Zn-MHA had a significant impact on the serum hormone indexes (Table 6). In general, the impact was associated with increasing supplement levels of Zn-MHA. Most obviously, serum FSH level in 25 mg/kg Zn-MHA group decreased (*P* < 0.05), while that in 75 mg/kg group increased (*P* < 0.01). The change of FSH level in serum was linear and quadratic (*P* < 0.001) with the supplement level of Zn-MHA in diet. Another thing worth noting is that P_4_ level in serum of breeders was also increased in the 75 mg/kg Zn-MHA group (*P* < 0.05). Serum T_3_ and E_2_ level in 25 mg/kg Zn-MHA group was lower than that in the ZnSO_4_ group (*P* < 0.05). No significant changes were observed in serum LH, PRL and T_4_ levels in all treatment groups.

**Table 6:** Effects of dietary Zn-MHA supplementation on serum hormone analysis of broiler breeders^a^.

**Items**	**Dietary Zn supplementation**				***P*** **-value**	
	**100 mg/kg ZnSO_**4**_**	**Zn-MHA (mg/kg)**				
		**25**	**50**	**75**	**Linear**	**Quadratic**
FSH (mIU/mL)	11.71 ± 1.72	9.22 ± 1.88^*^	11.29 ± 1.60	15.12 ± 0.95^**^	<0.001	<0.001
LH (ng/mL)	42.25 ± 15.25	31.66 ± 14.05	40.88 ± 7.44	43.46 ± 13.53	0.102	0.236
PRL (mIU/L)	397.57 ± 48.79	358.08 ± 58.54	390.92 ± 59.19	412.48 ± 103.44	0.223	0.482
E_2_ (pg/mL)	239.00 ± 29.01	174.29 ± 36.01^**^	236.63 ± 40.11	269.82 ± 44.36	0.001	0.003
P_4_ (pmol/L)	1253.93 ± 130.45	1195.88 ± 114.85	1330.61 ± 118.95	1417.09 ± 62.64^*^	0.001	0.007
T_3_ (nmol/L)	3.63 ± 0.58	2.52 ± 0.78^*^	3.31 ± 0.89	3.50 ± 0.36	0.028	0.070
T_4_ (nmol/L)	75.23 ± 20.81	57.49 ± 22.07	66.21 ± 25.49	82.77 ± 18.73	0.060	0.171

*Zn-MHA, Zinc methionine hydroxy analog chelate; FSH, follicle-stimulating hormone; LH, luteinizing hormone; PRL, prolactin; E_2_, estradiol; P_4_, progesterone; T_3_, triiodothyronine; T_4_, thyroxine*.

a *Data represent mean ± SD of 6 replicates each treatment*.

* *refers to P < 0.05 and*

** *refers to P < 0.01 between the control and experimental groups by independent-sample t test*.

### Effect of Zn-MHA on Reproductive Organ Development of Broiler Breeders

Compared with the ZnSO_4_ group, dietary supplementation of Zn-MHA did not affect the ovarian weight or the number of SYFs and LWFs in broiler breeders (Table 7).

**Table 7:** Effects of dietary Zn-MHA supplementation on ovarian development in broiler breeders^a^.

**Items**	**Dietary Zn supplementation**				***P*-value**	
	**100 mg/kg ZnSO_**4**_**	**Zn-MHA (mg/kg)**				
		**25**	**50**	**75**	**Linear**	**Quadratic**
Ovarian weight (g)	78.39 ± 1.06	76.25 ± 5.23	79.02 ± 4.00	81.02 ± 3.83	0.195	0.459
Number of SYFs	23.33 ± 1.53	20.33 ± 7.02	23.00 ± 8.00	23.67 ± 6.00	0.254	0.531
Number of LWFs	45.33 ± 2.08	37.33 ± 4.04	42.00 ± 3.00	44.00 ± 2.00	0.180	0.390

*Zn-MHA, Zinc methionine hydroxy analog chelate; SYF, small yellow follicles; LWF, large white follicles*.

a *Data represent mean ± SD of 3 hens each treatment*.

### Effect of Zn-MHA on Reproductive Axis Related MRNA Expressions of Broiler Breeders

**Figure 1:**
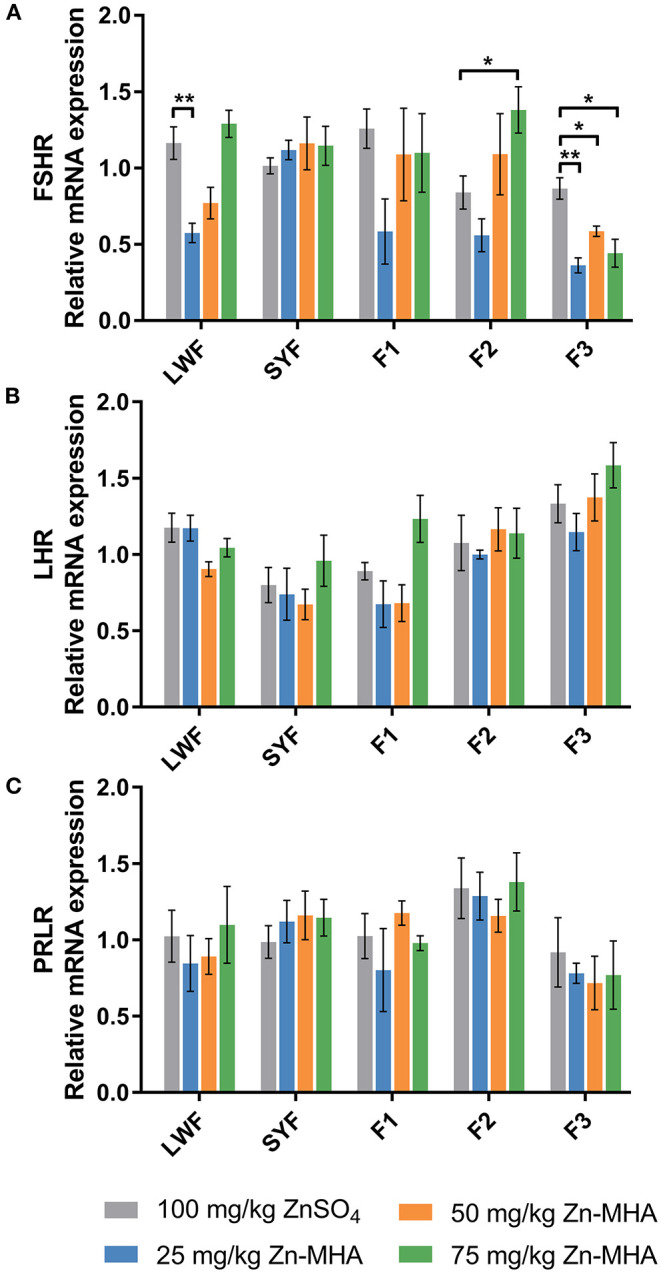
Effects of dietary Zn-MHA supplementation on the mRNA relative expressions of reproductive axis genes in broiler breeders. SYF, small yellow follicles (4 to 10 mm in diameter). LWF, large white follicles (2 to 4 mm in diameter). F1, the first largest one of preovulatory follicles. F2, the second largest one of preovulatory follicles. F3, the third largest one of preovulatory follicles. *FSHR*, follicle-stimulating hormone receptor; *LHR*, luteinizing hormone receptor; *PRLR*, prolactin receptor. **(A–C)** Data represent mean values of 3 hens each treatment as mean ± SEM. * refers to *P* < 0.05 and ** refers to *P* < 0.01 between the control and experimental groups by independent-sample *t* test.

Dietary supplementation of Zn-MHA affected mRNA expression of *FSHR* in LWFs, F2 and F3 (Figure 1A). The *FSHR* expression was down-regulated in 25 mg/kg Zn-MHA groups in LWFs compared to ZnSO_4_ group. However, no differences were observed between the 50 mg/kg and 75 mg/kg Zn-MHA diet-fed group and the ZnSO_4_ group. The *FSHR* mRNA expression was increased in the dietary supplementation of 75 mg/kg Zn-MHA in F2 (*P* < 0.05). However, there was a down-regulation in F3 between the ZnSO_4_ group and the 25 mg/kg, 50 mg/kg and 75 mg/kg Zn-MHA groups. No differences were detected for all the treatments of Zn-MHA in SYFs and F1.

The mRNA expression of *LHR* and *PRLR* measured in LWFs, SYFs and POFs was not affected by different Zn source (Figures 1B,C).

## Discussion

The role of organic Zn, especially amino acid chelated Zn, for human and animal health has been of constant interest (21). The concept of environmental protection and emission reduction is deeply rooted among the people, therefore, the studies on replacing inorganic Zn with organic Zn in broilers are of significant importance. In our study, supplementation of Zn-MHA to the broilers significantly affected FCR and egg weight. The results are consistent with the previous findings that the organic Zn has more benefits than inorganic Zn in promoting the laying performance of hens (22–24). The increase in FCR may be attributed to the effect of Zn on the increase in the related hormones such as P_4_ and FSH (16). The egg weight increased linearly with the increasing levels of Zn-MHA in diet. It is reported that an increase in hen's egg weight was observed of hens supplemented with organic Zn, which was similar to the present study (25). These improvements may be owing to the higher bioavailability of Zn-MHA, which can be better absorbed by animals and exert its important functions (26, 27). Different forms of dietary Zn have been reported to be efficiently deposited in eggs and transported to different tissues of offspring as embryos develop (28, 29). In addition, Stefanello et al. (30) found that using organic Zn instead of inorganic Zn did not exhibit a negative effect on FCR. In our study, we found that diets supplemented with 50 and 75 mg/kg Zn-MHA significantly improved FCR compared to the ZnSO_4_ group. Abd El-Hack et al. (31) reported that Zn-Met improved the feed conversion ratio in laying hens. However, Roshanzamir et al. (32) reported that dietary supplementation of organic Zn had no significant effect on the feed conversion ratio. The above inconsistencies might be due to the differences in Zn levels or experimental phases. The higher bioavailability of Zn-MHA could be responsible for the improvement of laying performance, which made hens make better use of Zn to secrete sex hormones. Moreover, the lower dose of Zn-MHA (75 mg/kg) appeared to have the same or even better effect than the 100 mg/kg inorganic zinc, which was attributed to the structural specificity advantage of the amino acid chelate.

The improvement of egg quality of hens by the dietary supplementation of Zn in the aged breeders is also a topic of concern. Age and nutrition are constant factors that affect egg quality. The decline of eggshell quality, such as eggshell thickness and eggshell strength, is a common phenomenon in aged laying hens, especially the expensive breeding eggs could bring greater economic losses (33). Results from previous studies have shown that supplementation of Zn in diets with either inorganic or organic forms had an impact on the egg quality (31, 34, 35). It is reported that adding Zn-MHA to the layer diet increased eggshell weight, eggshell thickness, eggshell strength and eggshell density (36). Also, partial or complete replacement of inorganic trace element compounds by organic trace element compounds has been proved to improve the eggshell quality and increase mineral deposition in eggs (37). These shreds of evidence also support our findings. A number of studies have shown that adding organic Zn to diets can significantly increase the Haugh unit score of eggs compared with inorganic Zn (15). Similar to these reports, we also detected some differences in egg quality between the Zn-MHA groups and ZnSO_4_ group, confirming that supplementation of Zn-MHA at the concentration of 75 mg/kg could not affect the egg quality. Also, we observed that lower doses of Zn-MHA (25 mg/kg) were not sufficient to meet the needs of breeders, resulting in reduced eggshell thickness.

Zn plays an indispensable role in the early meiosis of cells (38). Also, Zn functions in follicular rupture and cumulus expansion (39). These important effects may be directly related to the activity of Zn-dependent enzymes matrix metalloproteinases (40). In mammals, Zn enters the fetus through metallothionein, which is necessary for fetal growth and development (41). Zn also plays an important role in embryo development and egg hatchability, because the content of Zn in eggs is positively correlated with egg hatchability (3). It has been reported that “Zn sparks,” consisting of thousands of zinc vesicles, are triggered after the fertilization of a mammalian egg and are required to induce the development of the fertilized egg toward the embryo (42). Zhu et al. (43) reported that maternal dietary supplementation with the organic Zn improved hatchability. In general, Zn was reported to improve male sperm quality and is essential for the female reproductive system including embryogenesis and development (44). It is reported that Zn deficiency affected the reproductive function of rats (45, 46). In agreement with the results obtained by Favero et al. (47), we can detect some difference in egg fertility and hatchability between the supplementation levels of Zn-MHA and between Zn-MHA and ZnSO_4_. The hatching capacity, healthy offspring rates, and embryo mortality were not affected by treatment in the present study. Supplementation of 75 mg/kg Zn-MHA to diets of hens affects the quality of fertilized eggs. Also, no benefit was observed in reproductive performance in layers when dietary Zn level was over 150 mg/kg feed (48). One possible reason for this could be that the level of Zn in the basic diet was different and Zn-MHA had a higher bioavailability resulting in the supplementation of Zn in the diet thereby meeting the layer poultry requirement. More Zn enters into the body of hens to promote the secretion of reproductive hormones, which leads to the improvement of reproductive performance. And Zn also entered the eggs to promote the development of offspring.

Gonadotropins, such as FSH and LH, play a particularly important role in the course of follicular development and ovulation (49). FSH enhances the secretion of P_4_ by follicular granulosa cells through P450 side-chain cleavage in the preovulatory stage, so as to promote follicular development and maturation (50). LH, which has the same α-subunit as FSH, also has the function of promoting P_4_ secretion (51). Increasing levels of FSH and LH can also stimulate the secretion of E_2_ in follicles (52, 53). The raise of E_2_ could improve the sensitivity of the hypothalamic-pituitary axis to secrete more P_4_ (54). PRL secreted by the anterior pituitary is also important for the maintenance and secretory activity of the corpus luteum (55). The thyroid hormones constitute the endocrine system which has important functions in growth and energy utilization in birds (56). Therefore, these serum hormones have been considered as instructive indicators of laying performance (57). Root et al. (58) evidenced that Zn influenced FSH and LH activities in rats. It is reported that dietary zinc-methionine supplementation, at a level of 50 mg/kg DM, improved milk production and hormones in dairy camels (59). In the present study, we found that serum FSH and P_4_ levels were elevated with the increasing level of Zn-MHA supplementation. At the same time, we also detected such a trend in FCR, suggesting that dietary Zn-MHA could have a potential impact on the laying performance.

The increase of reproductive hormones in serum helps to increase the expression of related receptor genes in the ovary. FSH has been proved to stimulate ovarian *FSHR* expression (60, 61). Also, FSH mediates the expression of *LHR* by silencing Insig siRNA (62). The up-regulation of the above two receptors contributes to the binding of follicles to FSH and LH to promote follicular maturation (63). In the present study, the lower *FSHR* gene expression observed in broilers fed with 25 mg/kg Zn-MHA was consistent with the lower serum concentrations of gonadal hormones FSH and E_2_. While the increase in serum FSH level can be responsible for the up-regulation of *FSHR* mRNA in the 75 mg/kg Zn-MHA group. These hormones participate in the regulation of follicle development and pregnancy maintenance (64). Furthermore, we found that the changes of *LHR* and *PRLR* mRNA expression were consistent with the changes of corresponding hormones in serum, and there was no significant difference. The biochemical mechanisms for these changes need to be further studied.

## Conclusions

Overall, the present study drew the conclusion that replacing ZnSO_4_ (100 mg/kg) with a lower dose of Zn-MHA (75 mg/kg) in the diet of broiler breeders improved the laying and reproductive performance. Moreover, this increase was achieved by increasing levels of FSH and P_4_ in serum and up-regulated the expression levels of *FSHR* in the broiler ovary. In conclusion, it is suggested that Zn-MHA may be a potential substitute for inorganic zinc supplements in broiler breeders with higher bioavailability.

## Data Availability Statement

The raw data supporting the conclusions of this article will be made available by the authors, without undue reservation.

## Ethics Statement

The animal study was reviewed and approved by Animal Care and Use Committee of Hebei Agricultural University.

## Author Contributions

BY performed the experiments and drafted the manuscript. BY, JG, JJ, SL, and YH carried out the statistical analysis. ZF, GL, GZ, and YH helped the revision of this manuscript. ZF and GZ contributed to the supervision and guidance of the present study. All authors read and approved the final manuscript.

## Funding

This study was supported by the Hebei Province Technology R&D Program (20326617D and 19227305D).

## Conflict of Interest

The authors declare that the research was conducted in the absence of any commercial or financial relationships that could be construed as a potential conflict of interest.

## Publisher's Note

All claims expressed in this article are solely those of the authors and do not necessarily represent those of their affiliated organizations, or those of the publisher, the editors and the reviewers. Any product that may be evaluated in this article, or claim that may be made by its manufacturer, is not guaranteed or endorsed by the publisher.
